# Sustaining consistent condom use among female sex workers by addressing their vulnerabilities and strengthening community-led organizations in India

**DOI:** 10.1371/journal.pone.0235094

**Published:** 2020-07-01

**Authors:** Bidhubhusan Mahapatra, Monika Walia, Sangram Kishor Patel, Madhusudana Battala, Saradiya Mukherjee, Prachi Patel, Balakrishnan Subramanium, Yamini Atmavilas, Niranjan Saggurti

**Affiliations:** 1 Population Council, New Delhi, Delhi, India; 2 Catalyst Management Services, Bengaluru, Karnataka, India; 3 Vrutti, Bengaluru, Karnataka, India; 4 Bill & Melinda Gates Foundation, New Delhi, Delhi, India; Columbia University, UNITED STATES

## Abstract

**Introduction:**

Between 2014 and 2017, a program aimed at reducing HIV risk and promoting safe sex through consistent use of condoms sought to work through addressing social and economic vulnerabilities and strengthening community-led organizations (COs) of female sex workers (FSWs). This study examines if the program was effective by studying relationship between strengthening of COs, vulnerability reduction, and sustaining of consistent condom use behavior among FSWs.

**Methods:**

We used a longitudinal study design to assess the change in outcomes. A three-stage sampling design was used to select FSWs for the study. Panel data of 2085 FSWs selected from 38 COs across five states of India was used to examine the change in various outcomes from 2015 (Survey Round 1) to 2017 (Survey Round 2). The CO level program pillar measuring institutional development assessed performance of COs in six domains critical for any organization’s functionality and sustainability: governance, project management, financial management, program monitoring, advocacy and networking, and resource mobilization. Overall, 32 indicators from all these domains were used to compute the CO strength score. A score was computed by taking mean of average dimension scores. The overall score was divided into two groups based on the median cutoff; COs which scored below the median were considered to have low CO strength, while COs which scored above or equal to median were considered to have high CO strength. Multivariable regression modeling techniques were used to examine the effect of program pillars on outcome measures.

**Results:**

Analyses showed a significant improvement in the strength of the COs over time; percentage of COs having high strength improved from 50% in 2015 to 87% in Round 2. The improvement in CO’s strength increased financial security (Adjusted Odds Ratio [AOR]: 2.18, p<0.01), social welfare security (AOR: 1.71, p<0.01), and socio-legal security (AOR: 2.20, p<0.01) among FSWs. Further, improvement in financial security led to significant increase in consistent condom use with client among FSWs (AOR: 1.69, p<0.01) who were members of COs having high strength. Sustained consistent condom use was positively associated with young age (<30 years), ability to negotiate with clients for condom use, membership in self-help groups, high self-efficacy, self-confidence, and client solicitation in streets and brothels.

**Conclusions:**

Improving financial security and strengthening FSW led CO can improve sustained and consistent condom use. In addition, the program should focus on enhancing ability of FSWs to negotiate with clients for condom use, promote membership in self-help groups and target FSWs who are 30 years or older, and soliciting from homes to sustain consistent condom use across all FSWs.

## Introduction

While peer outreach, free or subsidized condoms, and the diagnosis and treatment of sexually transmitted infections (STIs) are the mainstay of HIV prevention programs [1–3], many studies/programs have underscored the importance of removing structural barriers that shape access and use of services and determine risk-aversion by vulnerable populations [4–7]. The Avahan program, funded by the Bill & Melinda Gates Foundation, transformed from a program focused on traditional HIV prevention approaches to one that sought to build community-led organizations (COs) of female sex workers (FSWs), men having sex with men (MSM), and transgender persons (TG), and in its final phase, address social and financial vulnerabilities as a prevention strategy [4, 8]. Evidence from the first two phases of Avahan (Phase 1: 2003–2008; Phase 2: 2009–2013) suggests the program was highly successful in reducing HIV infection by improving condom use, improving the up-take of HIV prevention services, and addressing violence, stigma, and cohesion [8, 9]. A mathematical modeling exercise indicated that by the end of the first phase of work focused largely on promoting consistent condom use, diagnosis, and treatment of STIs, about 42% of HIV infections were averted due to the Avahan program [10].

Recognizing that in order to sustain the gains of the first phase and address determinants of inconsistent condom use (like violence), a more concerted effort to strengthen community was given in Phase 2 of the program. During this phase, FSWs and other key population groups were mobilized into hotspot level groups (groups formed with 10–15 FSWs who usually solicit clients from the same place) in each district where program was being implemented. These groups then came together to form district level federations/organizations, known as COs [11–15]. It was envisioned that COs would take ownership of the program over time—as they are in the best position to understand the needs of the community. The COs were formally registered and functioned like any other non-profit organization. Each CO included about 2000 members, on average, and governed by a board elected by the CO members each year. Several rounds of training were provided during the second phase of intervention to the CO leadership team as well as the hotspot groups to build their capacity in program implementation and monitoring as well as resource mobilization.

Engaging COs in the implementation, the program was able to improve the sense of collectivization, self-efficacy for condom use and service utilization, self-confidence in availing various government health services, and negotiation skills for condom use with clients among FSWs [11, 12, 16–18]. Evidence from Avahan phase 2 research indicated that the improved collectivization among FSWs led to a better uptake of HIV prevention services and higher consistent condom use with clients relative to Avahan phase 1 [13, 19–21]. In addition, the evidences emerging from India and other developing countries by the end of Avahan Phase 2 indicated that the program needs to address the broader socio-politico-legal environment, as well as address individual vulnerabilities as a path to addressing the roots of risky behavior [22, 23]. For example, the Songachhi model, which started as a STI/HIV prevention initiative, has evolved over time to work with the community and sustain efforts while addressing the vulnerabilities of FSWs [24]. Similarly, a sex worker organization in Brazil worked to address different socio-economic vulnerabilities while implementing HIV programs [25]. Further, deliberations in various platforms suggested that capacity building of local community systems is key to the sustainability of health outcomes and in mobilizing funds from multiple sources [22]. Further, recent evidence suggests that 55% of FSWs were multidimensionally vulnerable, primarily due to lack of financial security and access to social welfare schemes [26]. Moreover, vulnerable FSWs were more likely to engage in risky sexual practices. Therefore, addressing vulnerabilities faced by community members can help in either sustaining or improving safe sex behavior.

**Fig 1:**
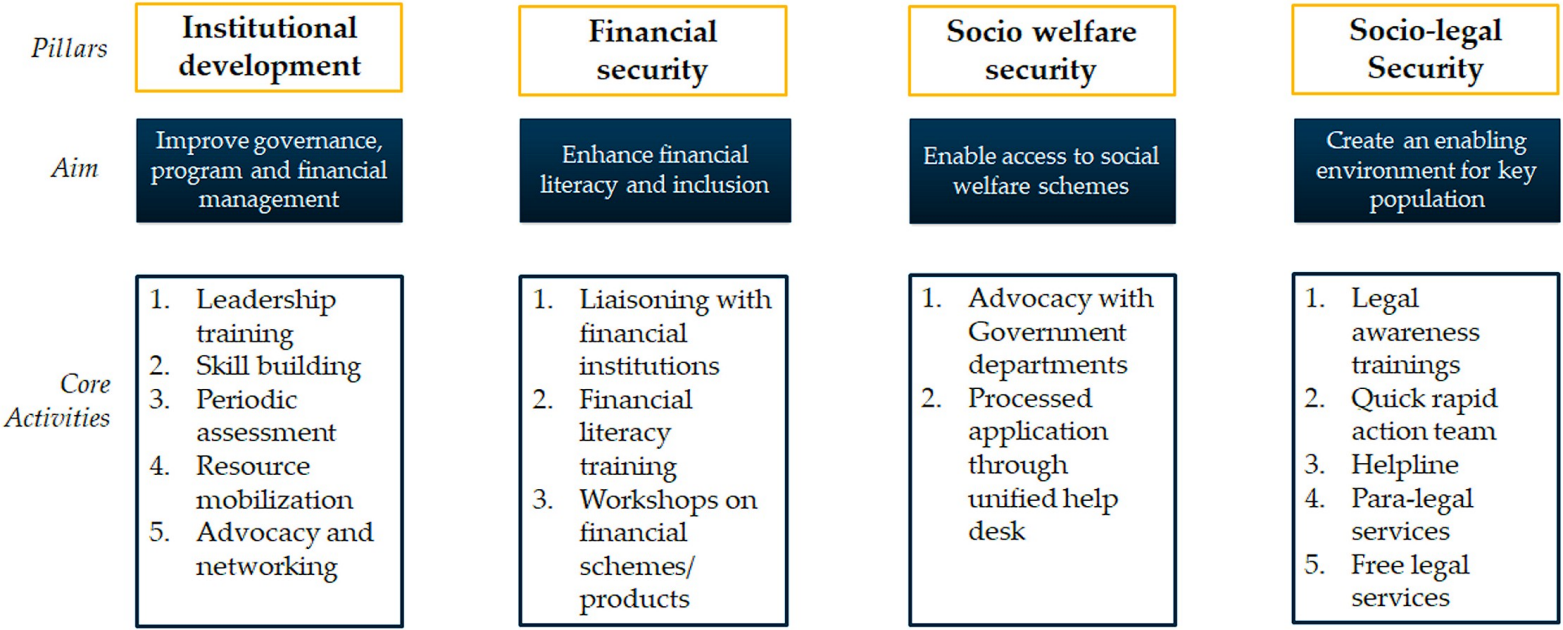
Program dimensions of Avahan 3 program and key activities implemented under each dimension.

In this backdrop, the final and third phase of the Avahan program (2014–2017) sought to sustain the gains made in the first two phases by investing on four key program pillars—institutional development, financial security, social welfare security, and socio-legal security (Fig 1). Institutional development, measured at the CO level, refers to the COs’ abilities in governance, program management, and resource mobilization. The other three dimensions, measured at individual level, reflect community members’ access to social welfare schemes (social welfare security), financial services (financial security), and para-legal services and awareness of rights (socio-legal security). Detailed description of the Avahan phase 3 intervention is provided in the next section. The program’s theory of change hypothesized that stronger institutions (COs) will have a greater capacity to generate funds, have ownership of the program, and implement a program that addresses the multidimensional (socio-economic and structural) vulnerabilities faced by CO members. Accordingly, the evaluation of Avahan Phase 3 sought to test two sets of interrelated hypotheses: (i) stronger institutions would serve to increase financial, social welfare, and socio-legal security; and (ii) improved financial, social welfare, and socio-legal security would enable and strengthen risk aversion and support safe behavior. The current study tests these two evaluation hypotheses and answers whether strengthening the capacity of COs reduces vulnerabilities among FSWs and thereby, contributes to the sustained consistent condom use behavior.

## Methods

### Study context

The study was conducted in five states of India: Andhra Pradesh, Telangana, Tamil Nadu, Karnataka and Maharashtra, where the Avahan-3 program was implemented. More than 120000 FSWs were registered under the Avahan program. According to the National Integrated Biological & Behavioral Surveillance (IBBS), the HIV prevalence among FSWs was highest in Maharashtra (7.4%) followed by in Andhra Pradesh/Telangana (6.3%), Karnataka (5.8%) and Tamil Nadu (1%).

### Intervention

The Avahan phase 3 program was implemented across 75 FSW and 12 MSM/TG COs and covered more than 160,000 FSWs and MSM/TGs across five states in India (Andhra Pradesh, Telangana, Karnataka, Maharashtra, and Tamil Nadu). While the grant was officially launched in April 2014, the roll out of standardized program components started in December 2014 and completed in all COs by July 2015. The program ended in December 2017. As indicated earlier, this phase of program focused on four key aspects: institutional development, financial security, social welfare security, and socio-legal security (Fig 1). While the institutional development focused on strengthening of COs, the other three aimed to empower FSWs by improving their access to entitlements, financial inclusion and creating a safer environment. The strengthening of the COs was done using various mechanisms. To start with, COs were assessed on their performance using participatory action methods on various components such as governance, project management, financial management, networking, linkages with other organizations, advocacy, and statutory requirements. Low performing component in each CO were identified and subsequently prioritized for strengthening. For example, if a CO performed poor in governance dimension, initially focus was on strengthening the governance system of that CO. Similarly, in a CO whose resource mobilization component was weak, emphasis was given on strengthening resource mobilization activities. The implementing partner of Avahan phase 3, Swasti, organized series of workshops addressing the identified components for performance. The trainings were implemented at multiple levels- state, regional, and CO level. The trainings were delivered by internal and external experts including FSWs with support from Swasti. The duration for each training was around 3–4 days.

For assessing the service needs of individuals, Swasti reached out to all FSWs in the CO catchment area through a Membership Engagement and Communication Tool (MECT). The MECT was administered through community outreach worker once every month for the first six months which were then aggregated to form a comprehensive information base. This helped in identifying the individuals, and clusters (geographical area of about 250–300 FSWs) where the program should prioritize. Using this, micro-plans were developed at outreach worker level. Two lines of leadership were built across the organizations with the improved ability to manage programs, and COs set up institutional mechanisms to address social welfare security, financial security, and socio-legal security of members. Unified Help Desks (UHDs) were established at each CO. The UHDs facilitated members in availing government entitlements and social welfare schemes and provided tools and guidance on financial planning and access to financial services. The UHD was created as a separated unit within COs and was managed by a UHD facilitator. The UHD had a dedicated helpline in which FSWs can contact and seek instant clarifications and help. The UHD facilitated all eligible members in the process of availing the benefits of various social and financial schemes. The UHD ensured that information about social welfare schemes along with necessary forms are provided to community. This helped CO members applying for multiple schemes without having to make multiple visits to government offices. In parallel, Swasti also organized financial literacy trainings at various levels (CO, outreach worker) to enhance FSWs knowledge on various financial products—saving accounts, investments, formal loan sources, and insurance mechanisms. Similarly, to improve access to social welfare schemes, communication materials on state specific schemes were prepared and distributed to FSWs. Further, UHD facilitated FSWs in verifying eligibility, suggesting relevant supporting documents, filling up forms, tracking of submitted forms and then supporting in case of any issues after submission. This support was enhanced by continuous engagement with the various government departments and financial institutions. A formal working relationship ensured minimal delay and rejections in applications processed through the UHD. One of the key components of Avahan program across all the three phases has been to improve the environment in which sex worker can practice sex work without any coercion. A crisis response system was built in each CO where each CO formed a crisis response committee to address any violence cases. The crisis response system consists of free legal aid and counseling to address physical or sexual violence faced by the members. Additionally, paralegal volunteers were identified in each CO who acted as community watchdog. These volunteers were trained in basic legal issues, including filing a First Information Report (FIR) with the help of district legal services authority. The UHDs helped FSWs in reporting any experience of violence to police and to seek redressal and resolution.

### Data

The study used data collected from 38 COs (of the 75 COs where program was implemented) across the five study states over two rounds in May-July 2015 (Survey Round 1, *hereinafter* referred as Round 1) and December 2017 (Survey Round 2, *hereinafter* referred as Round 2). The Andhra Pradesh and Telangana states were considered a single entity for sampling because they were one state when the study planned. The list of FSWs prepared by Swasti, served as the sampling frame for selection of FSWs. This list contained the name, unique identification number, and location details. A three-stage sampling process was used within each state to select the respondents. In the first stage, 10 COs were selected randomly within each state. In Maharashtra and Tamil Nadu, only 9 COs were available for selection; hence, all COs were included for the survey. In the second stage, three clusters (geographical area of about 250–300 FSWs) were selected within each CO. In the third stage, about 30–35 FSWs were randomly selected from each selected cluster. A minimum sample size of 873 (rounded up to 900) per state was estimated to detect a change of 10 percentage points in level of vulnerability faced by FSWs (lack of access to financial, social welfare, and socio-legal security) from a Round 1 value of 50% with 5% level of significance, 90% power, design effect of 1.7 and loss to follow-up rate of 40%. %. At Round 1, 3589 FSWs were interviewed; of which 2085 were re-interviewed at Round 2 of the survey. Therefore, all the analysis in this paper are based on a sample of 2085 FSWs who were surveyed in both rounds and had received the intervention prior to Round 2. Data was collected at both the CO and individual levels. CO level data measured performance and strength of COs, whereas individual data measured vulnerabilities and safe sex behavior. The CO level data was collected from CO board members or CO manager using a semi-structured tool and verification of records. The Individual data from FSWs were collected using a structured survey tool. All interviews were conducted by trained investigators proficient with verbal and written skills in the native language of each state.

### Ethics statement

The institutional review boards (IRBs) of Population Council and Sahara, Center for Residential Care and Rehabilitation, reviewed and approved the study procedure and tools. Prior to starting interviews, respondents were appraised of the study’s objectives, procedures, risks, and benefits associated with their participation. Written consent was obtained from respondents who could read and write, and for participants who could not, verbal consent was obtained in the presence of a witness (either program staff or fellow sex worker). All the interviews were held in a private location specifically hired for the survey or in a location convenient to the study participants.

### Measures

The key outcome measures for this study are *consistent condom use with clients and sustained consistent condom use*. FSWs were asked separately about the frequency of condom use with occasional (clients who visited FSWs occasionally for sex) and regular (clients who visited FSWs frequently for sex) clients in the past one month with response options of “always”, “most of the time”, “sometimes” or “never”. FSWs who used a condom “always” with both clients were considered consistent condom user; otherwise they were considered inconsistent users. Sustained consistent condom use was derived by comparing FSWs’ condom use behavior at Round 1 and Round 2 surveys. FSWs who used condoms consistently with all clients in both survey rounds were coded as 1 (i.e., had sustained consistent condom use), otherwise they were considered to have failed to sustain consistent condom use (coded as 0).

Three individual level program pillars, namely, financial security, social welfare, and socio-legal security were used as both outcome and predictor variables in the analysis. An aggregated score of *financial security* was created by adding single item questions (with response categories no/yes) on if FSWs had a savings account in the bank/post office, invested in financial products and services, invested in financial assets (such as gold, land and livestock), had an alternative source of income, and had not taken any loan from informal sources. The aggregated score (ranged from 0 to 6) was further categorized to ensure to make the findings program relevant where similar classification is used to identify vulnerable FSWs. The categorization of score into two categories was based on median level of the possible total score. Accordingly, FSWs who had a score above the median level were considered to have high financial security (coded as 1), otherwise they were coded as 0 (low financial security). Similarly, measures of *social welfare security* were created by combining information on whether FSWs had awareness about social welfare schemes (e.g. housing, pensions, ration, child education and girl child incentives), recipient of any benefit from any social welfare scheme, and possession of civic identity card such as Aadhaar card or voter identity card), possession of another identity card including ration card, income tax card, and owned a proof of residence such as passport, nativity certificate. All these variables were dichotomous with response categories 0/1. Measures of *access to socio-legal security* were created by combining an FSW’s experience of violence (physical and sexual), reporting of violence to an appropriate authority (police or paralegal cell), receipt of training in legal awareness, and intent to act collectively when violence was experienced. All these variables were dichotomous with response categories 0/1. Similar to financial security, aggregated scores on social welfare (ranged from 0 to 5) and socio-legal security (ranged from 0 to 4) were divided into two categories (low and high) based on the median split approach.

The CO level program pillar measuring institutional development assessed performance of COs in six domains critical for any organization’s functionality and sustainability: governance, project management, financial management, program monitoring, advocacy and networking, and resource mobilization. Questions and indicators for these domains were adapted from empirically tested tools such as community ownership and preparedness index tool [27]. Overall, 32 indicators from all these domains were used to compute the CO strength score. First, mean score of each domain was computed and then, an overall score was arrived at by taking the mean of the average domain score. The overall score was divided into two groups based on the median cutoff; COs who scored below the median were considered to have low CO strength, while COs who scored above or equal to median were considered to have high CO strength.

In order to identify predictors of sustained consistent condom use, several individual level socio-demographic and sex work-related information were collected from all respondent. Categorical variables such as literacy, marital status, living arrangement, and place of solicitation were recoded to ensure enough cell frequencies in each category. Age was asked as continuous variables and recoded into two categories: <30 years and 30+ years. Variables such as mobile for sex work, alcohol consumption in past 12 months, use of mobile phone for client salivation, practice of anal sex in last 12 months, experience of abuse or name calling in past six months and feeling safe to practice sex work at the current place were not recoded rather each item was included in the analyses in its original form. The study also asked FSWs if they are active members of self-help groups (SHGs) with response categories “no” and “yes”. SHGs are small collectives formed by group of women, usually 8–12 in number, who meets at regular interval and save a small amount of money in a pooled savings account. The savings are either used to give credit to SHG members on a rotation basis or to start any small-scale business. We also assessed if FSWs experienced any depressive symptoms in the week preceding the survey using the Center for Epidemiologic Studies-Depression scale-10 (CESD) scale [19]. FSWs whose CESD score was 10 or more were considered to have some level of depression, otherwise they were considered as having no signs of depression. In addition, we measured FSWs’ self-confidence and self-efficacy based on a series of statements which were read to respondents and their opinion was sought using a four-point Likert scale (completely confident, confident, somewhat confident and not at all confident). An FSW was considered to have self-confidence if she felt completely confident in one of the following action: (i) speaking her opinion in any training or CO meeting, (ii) talking to bank manager, (iii) talking to CO manager, (iv) talking to field workers or second-line leaders of the CO, (v) visit public distribution service (PDS) center to avail services and (vi) giving advice to fellow sex worker in accessing HIV services. Self-efficacy was assessed for based on their reporting indicators on condom use and service utilization. An FSW was considered to have self-efficacy if she reported completely confident to one of the following action: (i) able to insist on condom use with a client/partner even when (a) he got angry, (b) he offered more money for sex without condom, (c) FSW thought risk of disease was low, or (d) either FSW or partner had consumed alcohol; (ii) bought condom from a shop on her own; (iii) visited government health facility to seek reproductive health services even if health worker treated her badly; and (iv) visited government health facility to seek reproductive health services even if health worker knows her as a sex worker. These statements, used to assess self-efficacy and self-confidence, were taken from previous research implemented in India among FSWs [11].

### Statistical analyses

Univariate and bivariate analyses were conducted to present a profile of FSWs and prevalence of different outcome measures. A series of multilevel multiple logistic regression models were fitted to estimate the magnitude and significance of change in safe sex behavior and individual level program dimensions from Round 1 to Round 2. A multilevel model was fitted given the hierarchical nature of sampling process used in recruiting FSWs where FSWs were nested within COs and COs were nested with states. A multilevel analysis allows measure sources of variations within and across clusters. Moreover, multilevel models can correctly estimate standard errors enabling better inferential decision making. To test the first hypothesis, we fitted multilevel regression models with program pillars as dependent variables and an interaction term of survey round and CO strength as key predictor and adjusted for FSWs’ age, education, marital status, place of solicitation, mobility/migration for sex work, living alone or otherwise, exposure to earlier phases of Avahan program and state to which FSW belonged. Covariates included in the regression models included time varying in nature, that is, covariates’ values changed from Round 1 to Round 2. In addition to the interaction term, we also calculated the adjusted net change (in percentage) which was derived as a marginal probability from the regression model using Stata’s margin command. The command was run followed by the regression command to derive the marginal probability. The details about margin command can be found in the Stata manual [28]. To test the second hypothesis, we fitted separate models to measure the change over time in consistent condom use with clients in relation to the degree of change in program pillars. For this, consistent condom use was considered as the dependent variable with interaction term of survey round and program pillar as key covariate. Further, to understand simultaneous effect of change in CO strength and program dimension over time on consistent condom use, we used three-way interaction of survey round, program dimension and CO strength (at Round 2). Finally, we fitted a multiple logistic regression model based on the 2^nd^ round of survey data to identify individual socio-demographic and sex work-related predictors of sustained consistent condom use. For identifying predictors of sustained consistent condom use with clients, we first tested the bivariate relationship between individual covariates and sustained consistent condom use. Only those covariates which were associated with at least 10% level of significance were included in the final regression model. Results are presented in the form of percentages, odds ratios, and their corresponding 95% confidence interval (CI). All the analyses were performed using STATA 16.1 (StataCorp., TX, USA).

## Results

Respondents were about 35 years (Standard Deviation [SD]: 6.5) old, on average, with 58% having some level of formal education (Table 1). Nearly two-thirds of FSWs were currently married. While a little more than one-fifth of them were staying alone at the time of the Round 1 survey, only one-tenth stayed alone at the time of the Round 2 survey. Proportion of home-based FSWs reduced by eight percentage points from Round 1 to Round 2, whereas the proportion of brothel-based FSWs remained almost the same in the two rounds. The COs included in the study had been in opperation for an average of about eight years. The CO strength improved significantly from Round 1 to Round 2; 50% of COs had high CO strength at Round 1, which improved to 87% in Round 2.

**Table 1:** Individual- and CO-level characteristics of surveyed FSWs and community-led organizations, India.

Characteristics	Round 1	Round 2
	Mean (SD) or % (N = 2085)	Mean (SD) or % (N = 2085)
**Individual level (FSW) characteristics**		
Age, Mean (SD)	34.6 (6.5)	37.0 (6.5)
% with formal education	58.0	58.8
% currently married	64.0	63.6
% staying alone	21.5	10.7
**Place of solicitation**		
Home	51.3	42.9
Brothel	27.0	26.7
Street	21.7	30.4
% mobile for sex work	57.2	42.3
**CO level characteristics**	**N = 38**	
Average duration of FSW CO formation	7.6 (3.0)	
**CO strength**		
Low	50.0	13.2
High	50.0	86.8

Multifold improvements were noticed from Round 1 to Round 2 in all the three vulnerability measures (Table 2). For example, the odds having a high level of financial security was six times higher in Round 2 than in Round 1 (82% vs 50%, Adjusted OR [AOR]: 6.05, 95% CI: 5.00–7.33). Further, the improvement in all vulnerability measures was positively associated with the improvement in CO strength. With the improvement in CO strength from Round 1 to Round 2, financial security increased by 13 percentage points (AOR: 2.18, 95% CI: 1.29–3.68), social welfare security increased by 11 percentage point (AOR: 1.71, 95% CI: 1.17–2.50), and socio-legal security improved by 14 percentage point (AOR: 2.20, 95% CI: 1.48–3.29).

**Table 2:** Effect of change in CO strength on changes in financial security, social welfare security and socio-legal security among female sex workers (N = 2085).

Dependent variable	Round 1 (N = 2085)	Round 2 (N = 2085)	Round 2 vs Round 1	Net change owing to change in CO strength	
	%	%	AOR (95% CI)^£^	AOR (95% CI)^£^	Percentage point (95% CI)
**% FSWs having high:**					
Financial security	50.0	81.7	6.05 (5.00–7.33)	2.18 (1.29–3.68)	13.1 (4.1–22.0)
Social welfare security	37.2	71.4	6.34 (5.25–7.65)	1.71 (1.17–2.50)	11.0 (2.5–19.6)
Socio-legal security	57.8	85.1	5.36 (4.39–6.53)	2.20 (1.48–3.29)	13.8 (7.2–20.4)

^£^AOR: Adjusted Odds Ratio, CI: Confidence Interval; Derived from separate logistic regression models with covariates as age, education, marital status, place of solicitation, staying alone, mobility/migration for sex work, prior to exposure to Avahan program, CO strength, and survey state.

**Fig 2:**
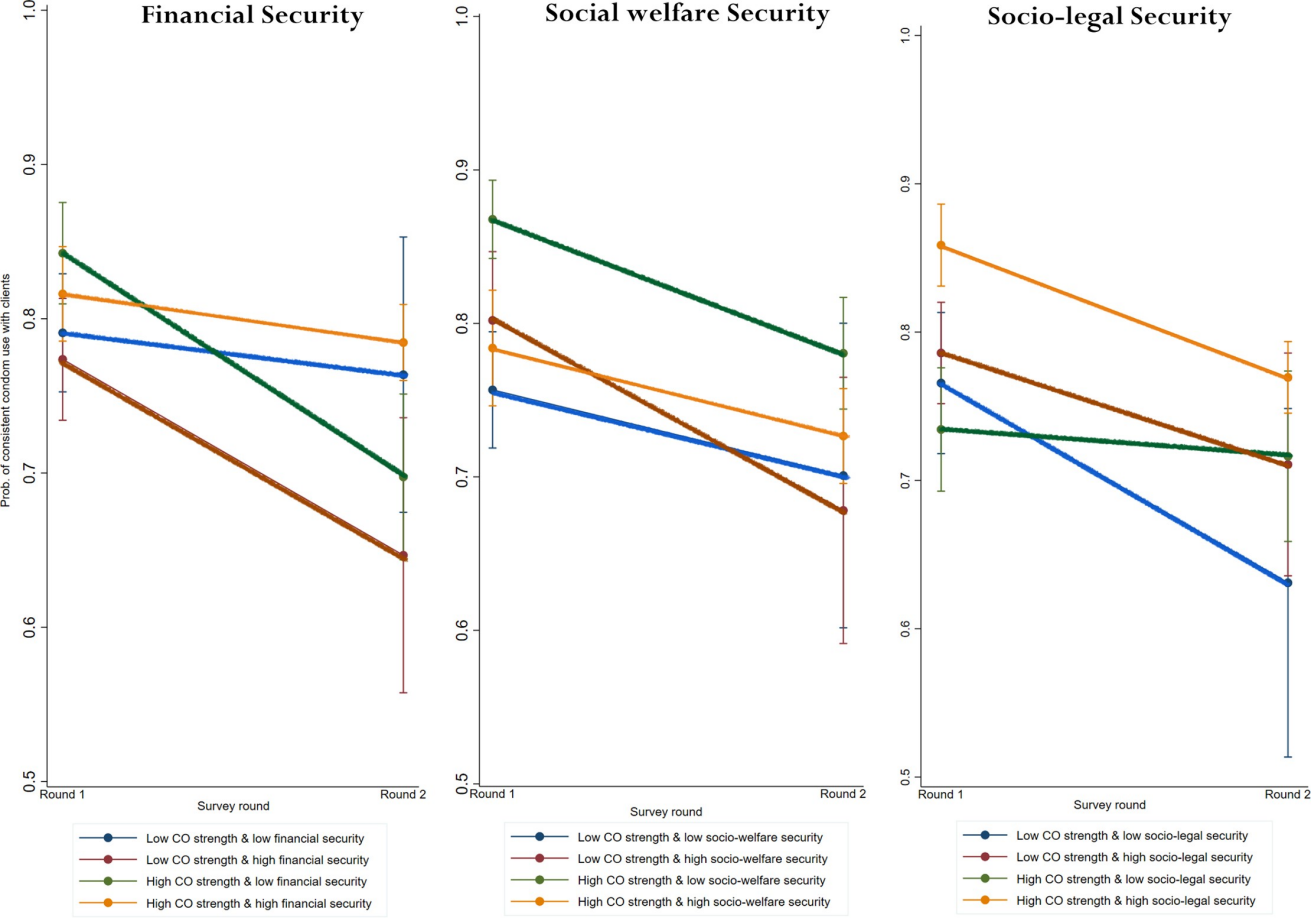
Percentage of FSWs using condom consistently with clients by program dimensions, CO strength and survey round.

The change over time analysis of consistent condom use with respect to individual program dimensions showed significant improvement in consistent condom use with clients among FSWs whose financial security improved over time and who were part of CO having high strength (AOR: 1.69, 95% CI: 1.13–2.52) (Table 3). Further, the odds of using condom consistently improves by five times with increase in financial security conditional to FSWs’ membership in a CO (Fig 2). That is, the improvement in financial security will have three times more impact on increasing consistent condom use if FSWs are members of CO having high strength than members in a CO having low strength.

**Table 3:** Change over time in consistent condom use with all clients with respect to change in individual program dimensions and CO strength among female sex workers (N = 2085).

	Low CO strength^€^		Change over time w.r.t change in program dimensions	High CO strength^€^		Change over time w.r.t change in program dimensions	Change over time w.r.t. change in CO strength and program dimensions
Survey Round	Round 1	Round 2	AOR (95% CI)^£^	Round 1	Round 2	AOR (95% CI)^£^	AOR (95% CI)^£^
**Overall**	79.6	66.7		79.5	77.8		
**Financial security**							
Low	82.2	76.6	0.47 (0.13–1.63)	78.8	71.8	**1.69 (1.13–2.52)**	**5.43 (2.01–14.68)**
High	72.7	57.9		80.1	78.9		
**Social welfare security**							
Low	79.0	72.3	0.61 (0.09–3.97)	80.1	77.2	0.90 (0.62–1.29)	2.02 (0.76–5.38)
High	86.7	61.0		78.7	78.0		
**Socio-legal security**							
Low	81.1	60.7	2.09 (0.64–6.83)	74.8	74.0	0.77 (0.51–1.15)	0.55 (0.21–1.44)
High	77.8	69.3		82.8	78.4		

^€^CO strength as measured at the time of Round 2 survey.

^£^AOR: Adjusted Odds Ratio, CI: Confidence Interval; Derived from logistic regression models with covariates as age, education, marital status, place of solicitation, staying alone, mobility/migration for sex work, prior to exposure to Avahan program, CO strength, and survey state.

The analysis on predictors of sustained consistent condom use suggests that a range of demographic, sex work-related and psychosocial factors are significant predict sustained consistent condom use among FSWs (Table 4). For example, FSWs who had high self-confidence were nearly two times more likely to sustain consistent condom use than those with low self-confidence (AOR: 1.91, 95% CI: 1.56–2.34). Similarly, FSWs having high self-efficacy (AOR: 2.74, 95% CI: 2.22–3.38), ability to negotiate with clients for condom use (AOR: 2.07, 95% CI: 1.61–2.66) and not having any depressive symptoms (AOR: 1.27, 95% CI: 1.02–1.59) were more likely to sustain consistent condom use than their counterparts. The odds of sustained consistent condom use were also higher among FSWs who were SHG members than non-members (AOR: 1.57, 95% CI: 1.28–1.91), and those practicing sex work at one place than being mobile (AOR: 1.28, 95% CI: 1.03–1.58). Further, the odds of sustaining consistent condom use with clients were higher among FSWs who solicited clients from brothels (AOR: 1.36, 95% CI: 1.02–1.82) and streets (AOR: 2.00, 95% CI: 1.51–2.64) than who solicited from homes. FSWs who felt safe practicing sex work at the current place and did not face any abuse or name calling in past six months were also more likely to sustain their condom use behavior.

**Table 4:** Predictor of sustained consistent condom use with clients among female sex workers with their socio-demographic and behavioral characteristics as predictor variables, India, 2018 (N = 2085).

Background characteristics	Number of FSWs	% FSWs who sustained consistent condom use	Crude OR (95% CI)^£^	Adjusted OR (95% CI)^£^
**Age**				
< 30 years	435	64.6	1.25 (1.00–1.55)	**1.35 (1.06–1.73)**
30+ years	1,650	59.4	Referent	Referent
**Literate**				
No	860	59.0	Referent	
Yes	1,225	61.6	1.11 (0.93–1.33)	
**Currently Married**				
No	760	58.7	Referent	
Yes	1,325	61.5	1.13 (0.94–1.35)	
**Lives alone**				
No	1,862	61.0	1.23 (0.93–1.62)	
Yes	223	56.1	Referent	
**Place of solicitation**				
Home	895	55.2	Referent	Referent
Brothel	556	58.6	1.15 (0.93–1.43)	**1.29 (1.02–1.64)**
Street	634	69.6	1.85 (1.50–2.30)	**1.83 (1.45–2.32)**
**Mobile for sex work**				
No	881	63.2	1.22 (1.02–1.46)	**1.28 (1.03–1.58)**
Yes	1,204	58.5	Referent	Referent
**Member of self-help group**				
No	1,044	54.2	Referent	Referent
Yes	1,041	66.8	1.70 (1.42–2.03)	**1.57 (1.28–1.91)**
**Consumed alcohol in last 12 months**				
No	1,495	62.0	1.25 (1.03–1.52)	1.13 (0.90–1.41)
Yes	590	56.6	Referent	Referent
**Used a mobile phone for client solicitation**				
No	442	61.5	Referent	
Yes	1,643	60.2	0.95 (0.76–1.17)	
**Able to negotiate with clients for condom use**				
No	359	43.2	Referent	Referent
Yes	1,726	64.1	2.35 (1.86–2.96)	**2.07 (1.61–2.66)**
**Had anal sex in last 12 months**				
No	1,740	61.2	1.20 (0.95–1.52)	
Yes	345	56.8	Referent	
**Faced abuse, name calling in past 6 months**				
No	1,821	62.0	1.63 (1.26–2.11)	**2.01 (1.47–2.74)**
Yes	264	50.0	Referent	Referent
**Felt safe while practicing sex work at current place**				
No	958	53.5	Referent	Referent
Yes	1,127	66.4	1.71 (1.43–2.04)	**1.29 (1.06–1.57)**
**Experienced any depressive symptoms**				
No	1,552	63.1	1.54 (1.26–1.87)	**1.27 (1.02–1.59)**
Yes	533	52.7	Referent	Referent
**Degree of self-confidence**				
Low	1,008	50.3	Referent	Referent
High	1,077	70.0	2.31 (1.93–2.76)	**1.91 (1.56–2.34)**
**Degree of self-efficacy**				
Low	744	45.7	Referent	Referent
High	1,341	68.7	2.61 (2.17–3.13)	**2.74 (2.22–3.38)**
** Total**	**2,085**	**60.5**		

^£^OR: Odds Ratio, CI: Confidence Interval

## Discussion

HIV prevention programs in India have made significant gains since the first HIV case was diagnosed in 1986. HIV prevalence has reduced from 0.48% in 2001 to 0.22% in 2017 [29]. Further, the rate of consistent condom use (with clients) among FSWs has increased by about 50% since 2001 [30, 31]. While these achievements are remarkable, the success of HIV prevention programs lie in sustaining positive behaviors (like consistent condom use). This study found that FSWs can sustain their consistent condom use behavior if vulnerabilities faced by them, particularly the financial one, are addressed. However, the reduction in vulnerability is dependent on the CO to which FSWs belong; strong COs are more responsive to, and address multiple vulnerabilities faced by, FSWs than their counterparts. The responsiveness of COs to address the needs of FSWs has helped them to adapt healthy and safe sex practices.

Institutional development through capacity building of community-led systems has been a key component of health system strengthening in several countries [32, 33]. Avahan COs have strengthened their capacity in governance, program management, financial management, resource mobilization, advocacy, and networking. Our findings show increase in CO strength from Round 1 to Round 2 that led to significant improvement in access to social welfare, financial, and socio-legal security. With stronger COs, the involvement of FSWs in designing and implementing of the program activities improved. While increased ownership led to smooth program implementation, complete ownership by COs is expected in the long run so that the programs will be sustained.

The study also found that improved financial security is a key driver in increasing consistent condom use. This is in line with previous studies which demonstrate that FSWs under poor financial conditions are more likely to engage in risky sexual practices [34–36]. Poor financial conditions have an inverse relationship with FSWs’ ability to negotiate for condom use with their clients [37]. The desire to earn money also forces FSWs to engage in many risky sexual activities, such as consumption of alcohol prior to sex or engaging in anal sex [38, 39]. Given the critical role of financial security among FSWs in shaping their sexual risk behavior, the Avahan Phase 3 program prioritized improving the financial condition of FSWs and was able to increase financial security by six-fold during the three years of intervention. The program specifically worked towards increasing financial literacy, helping in opening of savings accounts, and providing guidance on financial investments. Besides the focus on financial security, the program also focused on increasing access to social welfare security and socio-legal security. The national and state governments provide various benefits to individuals through a number of social welfare schemes. However, most FSWs are left out from receiving these benefits due to stigma and social discrimination associated with their profession. Given this, the COs had rounds of advocacy and networking meetings and consultations with government officials, and UHDs worked extensively to ensure eligible FSWs receive the benefits in which they are entitled. The Avahan phase 3 program further established a three-layered crisis response system to ensure that all violence related issues are addressed within 24 hours. Besides free legal aid, para-legal volunteers were recruited and trained, the 24/7 helpline was set up, and counseling was provided to support FSWs under duress. The comprehensive focus to improve financial security, social welfare security, and socio-legal security helped to address key vulnerabilities faced by FSWs.

The study highlighted that FSWs who have a high degree of self-efficacy and self-confidence and the ability to negotiate with clients to use condoms are more likely to sustain consistent condom use behavior than their counterparts. This is akin to empirical research which has shown that the positive influence of self-efficacy and self-confidence on condom use among FSWs [20, 37, 40, 41]. Past research in India has shown that having better financial security increases self-confidence, and hence, we believe FSWs could have increased their self-efficacy and confidence due to their improved financial security during the intervention period [16, 42, 43]. The study also found that being a member of SHG increases the likelihood of sustaining consistent condom use. This adds to the previous research which has shown a positive association between SHG membership and condom negotiation, better access to HIV prevention services, and condom use behavior [44, 45]. SHG provides a platform to marginalized women to empower themselves with information and opportunities that can enhance their economic condition [44]. Therefore, membership in SHGs helps FSWs become financially more secure, and hence, enables them to sustain condom use behavior over time.

The findings presented in the study should be interpreted considering the following limitations. First, there was no control group, hence, one should be cautious while attributing the changes in outcomes to the Avahan Phase 3 program, particularly for the gains made in access to social welfare security and financial security. At the time of the Avahan Phase 3 intervention, there were several initiatives by national and state governments that may have also contributed to the increased social welfare security and financial security. However, even with those initiatives in place, COs played a significant role in identifying needs and facilitating application forms. Second, we measured sustainability as consistent use of condoms in the past 30 days in both Round 1 and Round 2. FSWs could have skipped using a condom on one or more occasion between the survey rounds. Third, the study covered only the Avahan program area and there may be FSWs practicing sex work beyond the study area. Therefore, the interpretation of the study findings should be limited to the Avahan program area only. However, given that the Avahan program covered more than 70% of the FSWs in the five states, the findings of this study should hold true for the rest of the population. Fourth, there may be a certain extent of social desirability bias in some of the behavioral measures, such as condom use, due to their prior exposure to HIV prevention programs. Finally, the study did not collect baseline information before the program being rolled out in COs. Therefore, by the time Round 1 study was conducted some of the individuals may have actually benefited from their exposure to program which would have under-estimated the impact of Avahan Phase 3 program. Undoubtedly, the study would have benefited by having a baseline which could have helped in estimating a more realistic understanding about the impact of Avahan Phase 3 program.

The study provides important insights into the HIV prevention program in India and elsewhere. First, COs are highly capable of implementing programs and should be capacitated further so that they expand to other health domains. Moreover, with the ownership of the program, understanding of the needs of the community improves. In addition, the participation of community members in program design and implementation helps to address various implementation challenges and hence, enhances the probability of achieving various outcomes. Moreover, implementation programs through COs may be cheaper, as several tasks are undertaken by the community members voluntarily. An independent analysis of cost invested in Avahan 3 program suggest that the intervention only spent US$1.5 per FSW per year, which is comparatively cheaper than targeted HIV prevention programs implemented in India. Second, the key to sustaining consistent condom use behavior is to ensure financial security, access to social welfare security, and a system to redress crisis. Therefore, targeted HIV prevention programs should move beyond the traditional approach and include vulnerability reduction as a key program component. Addressing vulnerabilities coupled with targeted interventions may enhance the success rate of the program significantly. Third, SHGs can be a good platform to enhance the financial security of FSWs. Given the increasing emphasis on strengthening SHGs in the country, programs should encourage FSWs to be part of SHGs and take benefit of various economic opportunities.

## Conclusions

Consistent condom use behavior of FSWs can be sustained by addressing socio-economic vulnerabilities faced by FSWs and strengthening their organization. Moreover, improvement in the financial condition of FSWs is key to sustaining consistent condom use behavior. In addition, the program should focus on enhancing ability of FSWs to negotiate with clients for condom use, promote membership in SHGs and target FSWs who are 30 years or older, and soliciting from homes to sustain consistent condom use across all FSWs.
